# Vitamin A attenuates PFOS-induced neurotoxicity and alters early proximity patterns to conspecifics in zebrafish larvae

**DOI:** 10.3389/fnbeh.2025.1564694

**Published:** 2025-06-05

**Authors:** Peiyun Jiang, Jingyu Wang, Xiaoying Wang, Li Zou, Di Wu, Qu Xu, Yue Jiang, Mengmeng Yao, Qin Hong, Xia Chi

**Affiliations:** ^1^Women’s Hospital of Nanjing Medical University (Nanjing Women and Children’s Healthcare Hospital), Nanjing, China; ^2^Department of Pediatrics, The First Affiliated Hospital with Nanjing Medical University, Nanjing, China; ^3^The Fourth Affliated Hospital of Nanjing Medical University, Nanjing, China; ^4^Nanjing Medical Key Laboratory of Developmental Behavioral Pediatrics, Nanjing, China

**Keywords:** PFOS, social deficit, neurodevelopment, vitamin A, developmental behavior

## Abstract

**Introduction:**

Perfluorooctane sulfonic acid (PFOS), a persistent perfluoroalkyl substance with ubiquitous environmental distribution and bioaccumulative potential, has raised significant public health concerns due to its association with neurodevelopmental disorders. This study investigates vitamin A’s neuroprotective capacity against PFOS-induced toxicity, particularly focusing on social behavior deficits—a core phenotype of autism spectrum disorder (ASD).

**Methods:**

Zebrafish larvae were exposed to 1 μM or 5 μM PFOS (with/without 5 nM vitamin A co-treatment) from 24–144 hours post-fertilization (hpf). Control groups received 0.01% DMSO (vehicle) or 5 nM vitamin A alone. Developmental parameters (body length, heart rate), locomotor activity (total distance moved), and neurobehavioral endpoints (conspecific interaction) were quantified using automated tracking systems (ViewPoint ZebraLab). Neurochemical alterations were assessed through qPCR (dopaminergic genes) and AO staining (apoptosis).

**Results:**

PFOS exposure (5 μM) significantly increased inter-individual distance (IID) and reduced physical contact frequency during social interaction tests. Neurochemical analyses revealed concurrent dopamine transporter downregulation and apoptosis-related gene activation . Vitamin A co-treatment attenuated these effects.

**Discussion:**

Our findings demonstrate that PFOS disrupts early social neurodevelopment through dopaminergic dysregulation and apoptotic signaling, while vitamin A exhibits counteractive potential. this study elucidates the impact of PFOS exposure on zebrafish social behavior and brain development. while highlighting the neuroprotective potential of vitamin A against PFOS exposure, These findings have significant guiding implications for the development of public health policy and provide a scientific foundation for comprehending the neurotoxicity of PFOS and developing effective intervention measures.

## 1 Introduction

Perfluorooctane sulfonic acid (PFOS), a type of fluoride persistent organic pollutant (Domingo and Nadal, 2019), has been widely used in both industrial and consumer products (Ochoa-Herrera et al., 2016; Post, 2021). It is highly valued for its unique physical and chemical characteristics and is often used in the manufacture of food packaging (Zhang Y. et al., 2023), non-stick cookware (Kang et al., 2018), cosmetics (Jane et al., 2022), and waterproofing/lubricants (Arrieta-Cortes et al., 2017). Despite its utility, PFOS has raised significant health and environmental concerns due to its persistence and capacity for bioaccumulation. Research indicates that PFOS may pass through the blood-brain barrier and enter the brain (Hong et al., 2024; Xie et al., 2024). It may additionally influence nerve cell differentiation and proliferation, as well as disrupt the production and distribution of neurotransmitters (Sun et al., 2018; Yang et al., 2024). Environmental exposure to PFOS is therefore associated with a range of potential health risks (Holder et al., 2023; Oulhote et al., 2016; Ritscher et al., 2018). In response, governments have implemented measures to reduce its use and limit pollution caused by its disposal. Despite governmental efforts to limit PFOS emissions, they remain persistent in the environment and poses a long-term threat to human health (Fiedler et al., 2022; Southerland and Birnbaum, 2023; van der Veen et al., 2023).

Dopamine (DA), a monoamine neurotransmitter (Costa and Schoenbaum, 2022), plays a crucial role in maintaining neurotransmitter balance, regulating neuronal excitability and signaling, and supporting overall brain function (Hou et al., 2024; Li et al., 2022; Speranza et al., 2021). DA transmission influences behavior, cognition, and motor functions (Akiti et al., 2022; Kalyn et al., 2023; Ning et al., 2016). DA deficiencies have been linked to various conditions, including depression and attention deficit/hyperactivity disorder (Dong et al., 2023; Regan et al., 2022; Wu et al., 2023). The essential role and distinctiveness of dopaminergic neurons in the control of brain function have been elucidated by resent investigations into the regulatory function of midbrain dopaminergic neurons in the nervous system. Ju Wang et al. discovered that DA levels may influence social interactions through the gut microbiota of zebrafish (Wang J. et al., 2022). A Study conducted by Solie et al. on social behavioral representations in mice further demonstrated that different subsets of midbrain DA neurons modulate emotional cognition and motor function by targeting the striatum and cortex (Solié et al., 2022). In a study conducted by Wang et al. at Zhejiang University, they discovered that the neural mechanisms underlying social behavior are significantly influenced by dopaminergic activities (Wang et al., 2021). A study on the effects of cerebellar DA receptors on social behavior further demonstrated that DA receptors influence social abilities in mice by regulating synaptic plasticity (Cutando et al., 2022). This research underscores the importance of DA in cognitive and social functions, suggesting that targeting its pathways could be beneficial in mitigating social and neurological disorders.

In recent years, there has increased state focus on vitamin A levels in children and pregnant women due to growing awareness of health issues affecting these vulnerable populations. According to UNICEF approximately 140 million children worldwide are deficient in vitamin A, underscoring a significant global public health challenge (Chen et al., 2021). As a lipophilic vitamin, Vitamin A plays a crucial role in neural patterning and in promoting neurogenesis within the central nervous system (Shearer et al., 2012; Vo et al., 2023). Research indicates that insufficient levels of vitamin A impact the production and release of DA (Marie et al., 2022), a neurotransmitter that can lead to movement abnormalities (Carta et al., 2006). Retinoic acid, derived from Vitamin A, has been effective in halting DA neuron degeneration in rodent models of Parkinson’s disease (Esteves et al., 2015; Friling et al., 2009; Spathis et al., 2017; Yin et al., 2012). Furthermore, our previous research has demonstrated that vitamin A can reduce autism-like symptoms in zebrafish larvae exposed to VPA, an anticonvulsant and mood-stabilizing drug (Wang et al., 2024) Other studies have also reported that vitamin A relieves autism-like symptoms in rats and exerts a protective effect on neurodevelopment (Liu et al., 2022; Zhu et al., 2024). This findings suggest that Vitamin A may have therapeutic potential in mitigating neurodevelopmental issues and social impairments associated with PFOS exposure.

Based on the reviewed research, we hypothesize that PFOS exposure elevates DA levels and induces apoptosis, thereby potentially disrupting neurodevelopment and impairing social behaviors. This study aims to explore the impact of PFOS on zebrafish neurodevelopment, specifically assessing its effects on the DA system and apoptosis. We propose that vitamin A supplementation may mitigate these adverse effects by reducing elevated DA and apoptosis levels caused by PFOS, potentially decreasing neurological damage. Our findings are expected to enhance the understanding of PFOS-related neurotoxicity and support the development of evidence-based public health policies.

## 2 Materials and methods

### 2.1 Chemicals

Dimethyl sulfoxide (DMSO, ST038) was obtained from Biyotime. PFOS (CAS Number: 1763-23-1, purity 92.5%) was purchased from Dr. Ehrenstorfer™ Company, Germany. Acridine Orange (AO) Stain (CA1143, 1 mg/mL) was available at Solarbio, Vitamin A palmitate (CAS No: 79-81-2, purity ≥ 98%), N-Phenylthiourea (PTU) (CAS No: 103-85-5, purity ≥ 98%), and tricaine methanesulfonate (MS-222, purity ≥ 98%) were purchased from Sigma Aldrich.

Using DMSO as a solvent, PFOS was prepared at a concentration of 1 M. The undiluted PFOS was stored in a dark place at −20°C. The concentration of PFOS in the environment ranges from 0.14 nM to 5 μM (Calafat et al., 2019; Cordner et al., 2019; Yu et al., 2020; Zeng et al., 2019), so we chose 1 μM (low concentration group) and 5 μM (high concentration group) as our exposure doses. while the DMSO content in the control group was maintained at 0.01%. In this study, vitamin A at a concentration of 5 nM was selected for intervention based on the findings from previous studies (Wang et al., 2017).

### 2.2 Zebrafish compound exposure and toxicity

#### 2.2.1 Zebrafish and Husbandry

Adult zebrafish (3-month-old, TU) were obtained from Nanjing Yaoshunyu Biological Company. During the breeding phase, the zebrafish were housed in semi-recirculated water maintained at a pH of 6.5–7.5, hardness of 6–7, and a temperature of 25–26°C. Periodic cleaning of the fish tank was conducted as necessary. The fish were fed shrimp shells once in the morning and evening, and their feeding light was maintained at a 14-h light/10-h dark cycle. The breeding density was 6 fish/L. To generate embryos for the experiment, adult fish were propagated a couple of times a week, For each propagation Each time, adult fish were placed in a breeding tank equipped with an inner tank, using a 2:1 female-to-male fish ratio. Additionally, two-thirds of the system’s water was supplied. The following morning, at 8:00 a.m., the divider was taken down, allowing the zebrafish to engage in courtship and spawning behavior. The first 120 h post-fertilization (hpf) embryos were raised in incubators at 28°C. After 120 hpf, the embryos were transferred to a natural aquatic environment and fed three times daily with systematic water. All zebrafish experiments were approved by the Institutional Animal Care and Use Committee of Nanjing Medical University (IACUC-2309013).

#### 2.2.2 Zebrafish embryo exposure

According to OECD Test No. 236: Fish Embryo Acute Toxicity (FET) Test (Organisation for Economic Co-operation and Development. 2013)., the early development stage of zebrafish embryos is particularly sensitive to toxic substances (Kimmel et al., 1995), Therefore this study began to infect the embryos at 0 hpf. Healthy embryos were randomly selected and placed into 60-mmpetri dishes with 30 embryos in each dish. PFOS solution of 1 and 5 μM with or without 5 nM VA were added, respectively, for toxic exposure. The exposure duration was 0–144 hpf.

#### 2.2.3 Assessment of general developmental toxicity of zebrafish

Randomly divide healthy embryos into three groups, with 30 embryos in each group. Add 0.01% DMSO, 1 and 5 μM PFOS to the embryos in the three groups respectively. Mortality and hatching status were recorded every 24 h. Dead juvenile fish or embryos were promptly removed. To observe spontaneous tail-wagging movement, zebrafish embryos were incubated for 24 h. Each embryo was gently placed into a 24-well plate containing embryo culture medium. At 25°C, the spontaneous tail-wagging movement of each embryo was recorded for 1 min using a stereomicroscope. Tail-wagging was defined as a complete lateral bend of more than 30° along the body axis. Eight embryos from each group were analyzed, and the recorded videos were reviewed by two blind observers to ensure consistency. The embryo heartbeats were assessed after 48 hours. The embryos were placed under a stereomicroscope with their back facing upward. Heart rates were recorded over a 20-s interval, with 6 embryos analyzed per group. All embryos were independently evaluated by two researchers during the observation.

At 72 hpf, zebrafish larvae from the various infected groups were randomly selected. A Leica microscope was used to capture images and measure both the body length and head area of zebrafish. 10 larvae were recorded in each group.

### 2.3 Zebrafish behavior

The motor behavior of zebrafish was assessed at 144 hpf. At this time, the motor behavior of zebrafish was relatively stable, and their responsiveness to environmental changes and external stimuli was elevated, which could better reflect the influence of poisoning on movement (Correia et al., 2024; Leite et al., 2025; Santos et al., 2024; Xu et al., 2024).

#### 2.3.1 Open field test

Open field experiment was employed to assess the motor ability and novelty-induced responses response of larvae. In this experiment, fish are placed in a novel habitat (5 cm × 5 cm × 3 cm). A nested square, measuring 2.5 cm per side—50% of the original square’s side length—was constructed at the geometric center. The nested square defines the central area of the open field, whereas the remaining part constitutes the edge area. Curious larvae explored and acclimatized to their surroundings, whereas anxious and nervous fish will stayed or migrated closer to the edge of the module. In this experiment, we used zebrafish larvae that were 144 hpf and were housed in two specially designed modules. Each module was filled with 2 mL of system water, and then inserted into the track tracker (Viewpoint Zebralab, French). The apparatus was maintained at 28°C under standard lighting conditions while using a juvenile fish tracking software for monitoring. Subsequently, two regularly developing larvae were randomly selected, and one larva per acrylic module was transferred to the culture tank Following a 30-min environmental adaption period, larval behavior was observed for an additional 30 min. During this observation period, the duration and distance of the larvae movement within the module were recorded every 10 min. Movement patterns of the larvae were compared across groups, with 10 zebrafish larvae tested in each group.

#### 2.3.2 Assessment of larval proximity patterns

We examined the social behavior of zebrafish larvae at 144 hpf. Twelve typically developed larvae were randomly selected and placed on a six-well plate with two fish and 2 mm of system water per hole. Following a 30-min adaption period, a 30-min test of behavior was conducted. The average social distance was defined as the average distance (in mm) between the body centers of two fish during a 30-min observation period. The contact time ratio was calculated as the proportion of time during which the distance between the body centers of the two fish was less than or equal to the average body length of one fish. The proportion of contact time is equal to the contact time divided by the total time. Twelve zebrafish larvae were tested in each group.

#### 2.3.3 Group behavior

A total of 10 larvae, each 6 days old, were selected from the culture tank. 5 mL of system water was added to the circular module (10 cm in diameter), which was then carefully inserted into the track tracker. Following a 10-min period of environmental adjustment, larval behavior was observed for 30 min, during which fish behavior norms were examined. Among the recorded metrics were the minimal distance (NND, mm) and inter-individual distance (IID, mm) between two fish. The NND was defined as the minimum distance (in mm) between any two fish in a group. The IID was defined as the average paired distance (in mm) between all fish in the area, reflecting the cohesion of the group. To ensure the absence of chemicals in the water, the water in the container was replaced after recording each group of fish during the behavioral experiment. Each group consisted of 10 zebrafish larvae.

### 2.4 AO staining

Zebrafish embryos were cultured with 0.003% PTU embryo culture water, there are 10 larvae in each group treated with inhibition of pigment for at least 72 h, as described in our previous study where we performed an AO staining experiment at 96 hpf (Zhu et al., 2021). A total of 10 juvenile zebrafish were randomly selected from each group, treated with 0.01% tricaine methanesulfonate, and transferred into an EP tube containing 1.5 mL AO Stain (5 μg/mL). The zebrafish were stained at room temperature for 20 min in the dark. Following three washes with double-distilled water, apoptotic cells in the zebrafish brain tissue were observed and recorded using a body fluorescence microscope with an excitation light wavelength of 488 nm.

### 2.5 Dopamine content measurement

Zebrafish larvae at 168 hpf were collected, and 30 individuals from each exposure group were randomly selected for the quantification of DA levels. Whole zebrafish larvae were homogenized in ice-cold PBS, and DA was extracted from the whole-body lysate following a previously described method (Huang et al., 2022). DA levels were then measured using a zebrafish DA ELISA kit manufactured by Enzyme Free Company, in accordance with the manufacturer’s instructions. The DA content is reported in μg/mgprot.

### 2.6 Gene expression analysis

Total RNA was extracted from whole zebrafish larvae (*n* = 30 per group) using Trizol reagent (Vazyme, R411-01) following homogenization with a mechanical homogenizer (15,000 rpm, 30 s). RNA was purified via chloroform–isopropanol precipitation, and RNA purity was measured using a NanoDrop 2000 spectrophotometer (A260/A280 ratio: 1.8–2.0). cDNA was synthesized from 1 μg of RNA using HiScript III RT SuperMix (Vazyme, R223) in a 20 μL reaction volume. qPCR was performed on a Bio-Rad CFX96 system with ChamQ Universal SYBR qPCR Master Mix (Vazyme Biotech, Q711-02) under the following conditions: 95°C for 30 s, followed by 40 cycles of 95°C for 10 s, and 60°C for 30 s. Each 20 μL reaction contained 10 μL SYBR mix, 0.4 μM primers, and 1 μL cDNA template (technical triplicates per sample). Relative gene expression was calculated using the 2^–△△^Ct method, with *gapdh* as the internal control gene validated for stable expression across experimental groups [one-way analysis of variance (ANOVA), *P* > 0.05] and consistent with previous zebrafish studies (Liu et al., 2018). Primer sequences are listed in Table 1. The 2^–△△^Ct method was used to assess the target gene’s relative expression using *gapdh* as the internal parameter.

**TABLE 1:** Primers used in qPCR validation.

Primers	Forward sequence (5′–3′)	Reverse sequence (5′–3′)
*gapdh*	GATACACGGA GCACCAGGTT	CAGGTCACATA CACGGTTGC
*th1*	GACGGAAGATGAT CGGAGACA	CCGCCATGTTCC GATTTCT
*th2*	CTCCAGAAGAGAA TGCCACATG	ACGTTCACTCT CCAGCTGAGTG
*dat*	AGACATCTGGGAA GGTGGTG	ACCTGAGCATCAT ACAGGCG
*drd1*	ACGCTGTCCATC CTTATCTC	TGTCCGATTAAG GCTGGAG
*drd2a*	TGGTACTCCGGA AAAGACG	ATCGGGATGGGT GCATTTC
*drd3*	ATCAGTATCGACA GGTATACAGC	CCAAACAGT AGAGGGCAGG
*drd4a*	GCCTCTTTCCCA TCTCACAG	CTCAGACACAC CAGCACGTT
*bax*	GGCTATTTCAACCAG GGTTCC	TGCGAATCACCA ATGCTGT
*caspase-3*	CCGCTGCCCA TCACTA	ATCCTTTCACG ACCATCT
*caspase-8*	CCAGACAAT CTGGATGAACTTTAC	TGCAAACTGCTTT ATCTCATCT
*caspase-9*	CTGAGGCAA GCCATAATCG	AGAGGACATGGGA ATAGCGT
*gfap*	AGCTACATCGAG AAGGTGCG	TTTGAGAGTGCCG AGGTCTG
*mbpa*	TGGCTTGGATTG TATGCCCT	GCTCCCACTGACT CTTTGTCC
*bdnf*	CTCACGGACACT TTCGAGCA	TCAACGTCCTTCG AGTCTGC
*elavl3*	AGAAGTGTGA GGCGGGAATG	CTGGGGCAAGA TTTCGGGAT

### 2.7 Statistical analysis

In this study, GraphPad Prism version 9 (GraphPad Software) was used to analyze the obtained data. Sample sizes (n values) for each experiment are presented in the figure legends. The final calculated value was expressed as the mean ± standard error (SEM). The control and exposure groups were assessed using one-way ANOVA and Tukey *post-hoc* test. ANOVA was used to obtain *P*-values, which were analyzed to determine their significance. The *P*-values and were considered statistically significant when *P* < 0.05.

## 3 Results

### 3.1 Effects of PFOS exposure on survival and general development of zebrafish embryos/larvae

We evaluated the impact of PFOS on the survival rate and hatching success of zebrafish, as well as its effect on spontaneous movement and heart rate. Additionally, we assessed potential morphological changes by measuring the body length and head area of exposed embryos in comparison to the control group. We initially monitored the survival and hatching rates, starting from 0 hpf. We observed that PFOS exposure did not affect either the survival (S. 1A) or hatching rates (S. 1D) of the zebrafish when compared to the control group. Under a microscope, we then documented the natural movements and heartbeat frequencies of the embryos. We applied this concentration to track the stimulation of zebrafish embryos and observed no difference in the stimulation of the embryos’ heartbeat (Supplementary Figure S1C) or spontaneous movement (S. 1B) compared to the control group. Simultaneously, we measured the head area (Supplementary Figure S1E) and body length (Supplementary Figure S1F) of the zebrafish and individually compared these metrics with those of the control group. We discovered no discernible difference between the head area (Supplementary Figure S1G) and body length (Supplementary Figure S1H) when compared with the control group. After accounting for abnormalities in mobility and other factors, we conclude that the PFOS concentrations used did not significantly impact the gross development of the zebrafish.

### 3.2 PFOS exposure induces anxiety-like behavior and alterations of larval proximity patterns

**FIGURE 1:**
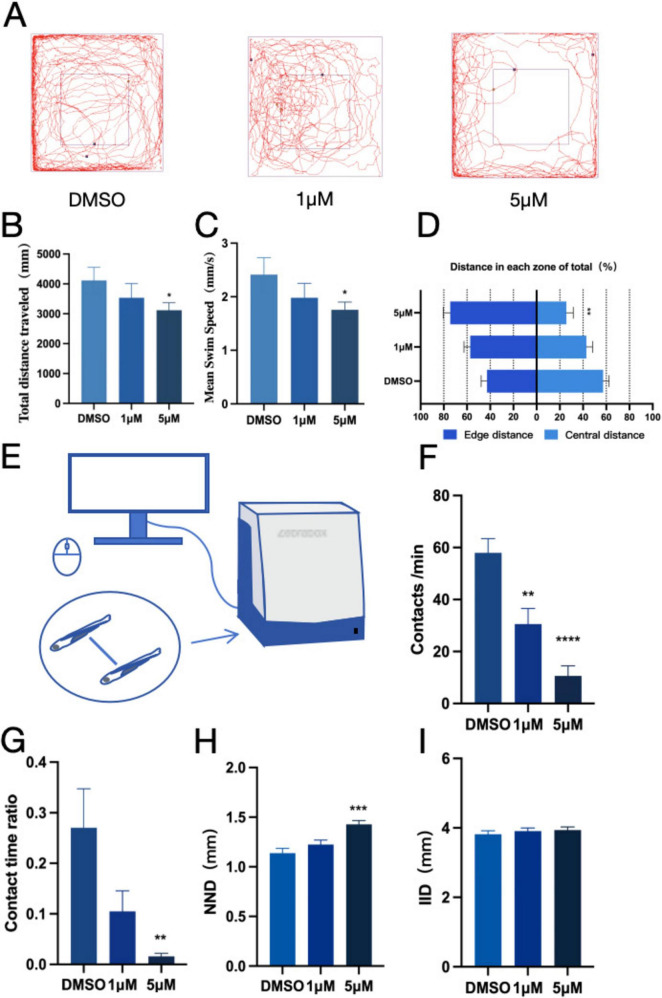
PFOS exposure causes anxiety-like behavior and social disturbance in zebrafish. **(A)** Open field path map of zebrafish. **(B)** Total distance of zebrafish open field movement (*n* = 10). **(C)** Average speed of zebrafish open field (*n* = 10). **(D)** The proportion of distance between central and marginal areas of zebrafish (Edge area distance ratio = outer distance/total distance, central area distance ratio = inner distance/total distance) (*n* = 10). **(E)** Diagram of a zebrafish social interaction experiment. **(F)** Zebrafish contacts per min (*n* = 12). **(G)** Zebrafish contact duration ratio (*n* = 12). **(H)** Zebrafish Minimum distance between two fish (*n* = 10). **(I)** Average distance between two zebrafish (*n* = 10). These data are expressed as mean ± SEM. *P < 0.05, **P < 0.01, ***P < 0.001, ****P < 0.0001 versus DMSO group.

When zebrafish migrate to a new habitat, they typically exhibit exploratory behaviors, such as migrating toward the center of the new habitat. To explore the anxiety-like behavior of zebrafish, an open-field experiment was conducted, examining parameters including movement track, time, and distance. Results The movement trajectory of zebrafish exposed to PFOS exhibited significantly altered patterns (Figure 1A), along with a considerable reduction in both total movement distance (Figure 1B) and the average speed (Figure 1C). Furthermore, we observed a significant decrease in movement within the center of the new habitat in zebrafish exposed to PFOS, with a noticeable increase in peripheral locomotion (Figure 1D). The findings demonstrated that PFOS impaired the exercise intensity and efficiency of zebrafish, with less time spent in the central zone indicating a decreased ability of infected zebrafish to adapt to the new environment and an increased anxiety-like behavior. Next, we assessed the social interaction capacity of zebrafish (Figure 1E). PFOS-exposed larvae exhibited reduced proximity duration and increased IID (Figure 1G), fewer conspecific contacts (Figure 1F), and significant alterations when exposed to 5 μM PFOS in comparison to the control group. Upon comparing the preferences of infected and control zebrafish groups, we determined that zebrafish in the control group were more likely to remain in close proximity to conspecifics, whereas both NND (minimum proximity distance) (Figure 1H) and IID (average distance between two fish) (Figure 1I) of zebrafish in the infected group increased. This suggests that exposure to PFOS altered fish cohesion and preference, as well as increased the distance between individual fish. Thus, we deduced that zebrafish exposure to PFOS resulted in mobility impairment, which most likely affected their social interaction behavior.

### 3.3 Effects of PFOS exposure on neurodevelopment and expression of dopamine-related genes

**FIGURE 2:**
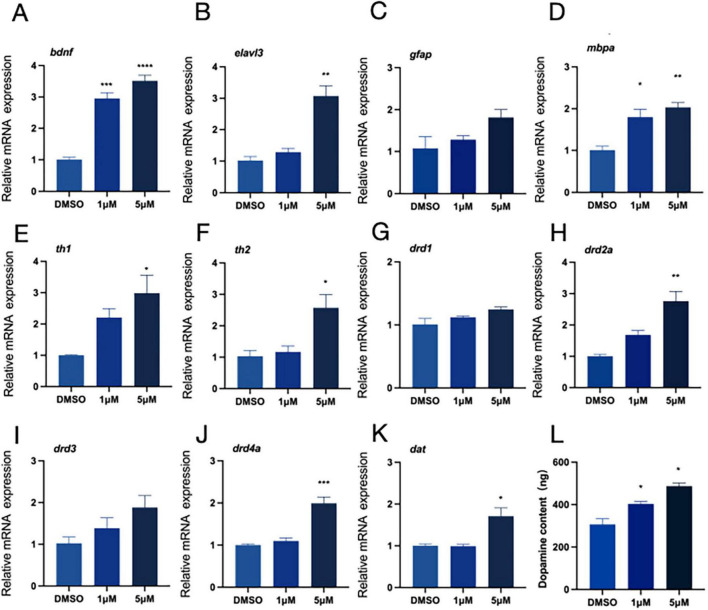
Neurodevelopment and relative expression of dopamine-related genes. RNA was extracted from at least 30 fish each time. **(A–D**) Expression of neurodevelopment-related genes (four genes, *bdnf, elavl3, gfap* and *mbpa* were detected). **(E–K)** The relative expression of genes related to dopamine pathway *(th1, th2, drd1, drd2a, drd3, drd4a*, and *dat* were detected). **(L)** Dopamine content (test and calculate according to ELISA kit). These data are expressed as mean ± SEM. *P < 0.05, **P < 0.01, ***P < 0.001, ****P < 0.0001 versus DMSO group.

Our earlier research suggest that exposure to PFOS might alter zebrafish proximity-based interactions. To further investigate whether PFOS exposure affects the neurological system of zebrafish and assess its impact on the dopaminergic system, we selected relevant genes using real-time quantitative fluorescence PCR (qRT-PCR) technology. We examined early neurodevelopment-related genes (*bdnf, elval3, gfap*, and *mbpa*) (Figures 2A–D), DA receptors (*drd1, drd2a, drd3*, and *drd4a*) (Figures 2G–J), the DA signaling pathway *(th1, th2*) (Figures 2E,F), and the DA transporter (dat) (Figure 2K). All of these genes exhibited increased expression following PFOS exposure. Notably, there was a dose-dependent up-regulation of the expressions of the genes *bdnf, elavl3, mbpa, dat, drd3, drd4a, th2*, and *drd1. Additionally, DA content measurement revealed that PFOS exposure altered the zebrafish dopaminergic system, with increased DA levels observed* (Figure 2L).

### 3.4 Changes in dopamine system induced by PFOS exposure may also affect apoptosis

**FIGURE 3:**
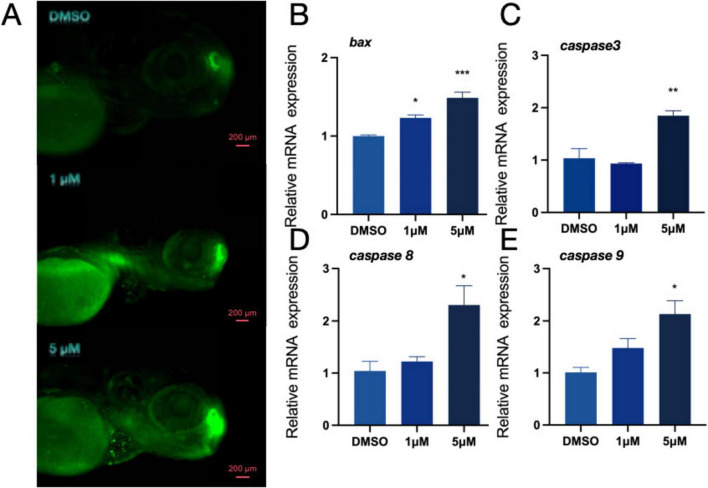
Effect of apoptosis pathway in zebrafish. **(A)** AO staining apoptotic corpuscles of zebrafish. **(B–E)** Relative expression of genes related to apoptosis pathway in zebrafishbdnf (*bax, caspase-3, caspase8* and *caspase-9* were detected) (*n* = 10). These data are expressed as mean ± SEM. *P < 0.05, **P < 0.01, ***P < 0.001 versus DMSO group.

Toxic byproducts of DA metabolism have the potential to cause cell damage and trigger programmed cell death. Therefore, after observing the PFOS-induced changes in the dopaminergic system, we investigated whether PFOS exposure could lead to apoptosis. We stained zebrafish larvae with AO (Figure 3A) and photographed them with fluorescence microscopy. An increase in granular green plaques (indicative of apoptotic cells) was observed in the head of juvenile zebrafish, Additionally, expression levels of apoptosis-related genes *bax, caspase3, caspase8* and *caspase9* were also up-regulated (Figures 3B–E), suggesting that PFOS exposure induced apoptosis in juvenile zebrafish. These findings suggest that PFOS may cause apoptosis of zebrafish.

### 3.5 Vitamin A alleviates PFOS-induced neurotoxicity by improving the dopaminergic system

**FIGURE 4:**
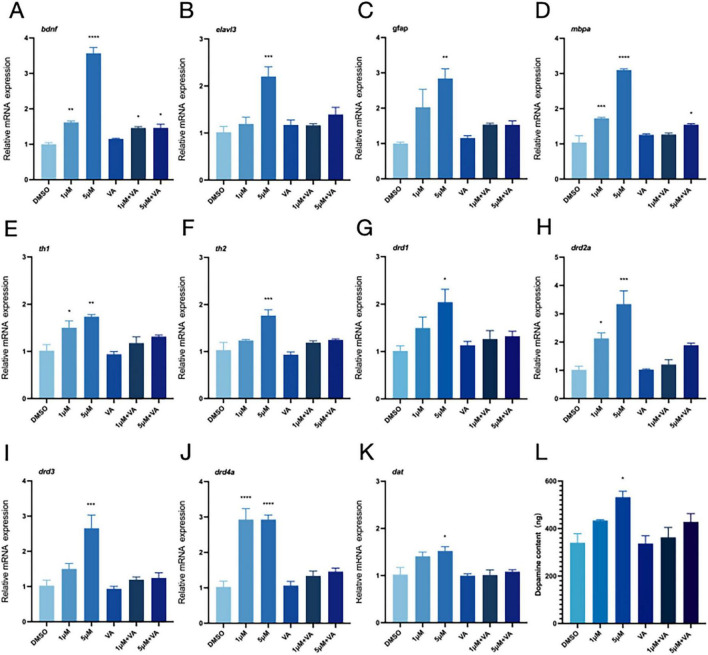
Vitamin A improves neurodevelopment and the relative expression of dopamine-related genes. DMSO was used as the control group in this experiment. The 1 μM group represented the exposure group with a PFOS concentration of 1 μM, and the 5 μM group represented the exposure group with a PFOS concentration of 5 μM. “VA” refers to the vitamin A group; 1 μM + VA denotes the vitamin A intervention group with a PFOS concentration of 1 μM, and 5 μM + VA denotes the vitamin A intervention group with a PFOS concentration of 5 μM. **(A–D)** Expression of neurodevelopment-related genes (four genes, *bdnf, elavl3, gfap* and *mbpa* were detected). **(E–K)** The relative expression of genes related to dopamine pathway (*th1*, *th2, drd1, drd2a, drd3*, *drd4a* and *dat* were detected). **(L)** Dopamine content (*n* = 30 per group). These data are expressed as mean ± SEM. *P < 0.05, **P < 0.01, ***P < 0.001, ****P < 0.0001 versus DMSO group.

We demonstrated that vitamin A might somewhat lower the expression levels of genes linked to neurodevelopment (*bdnf, elval3, gfap, and mbpa*) to alleviate PFOS-induced abnormalities in the dopaminergic system abnormalities (Figures 4A–D). The effects of PFOS exposure on zebrafish cerebral development were mitigated, as was the up-regulation of genes associated with the dopaminergic system (Figures 4E–K) and DA content (Figure 4L).

### 3.6 Vitamin A alleviates apoptosis induced by PFOS

**FIGURE 5:**
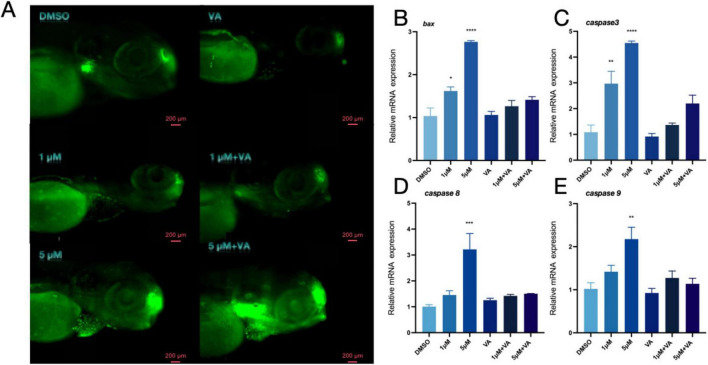
Vitamin A improves apoptosis of zebrafish. **(A)** AO staining apoptotic corpuscles of zebrafish. **(B–E**) Relative expression of genes related to apoptosis pathway in zebrafish (*bax, caspase-3, caspase8* and *caspase-9* were detected) (*n* = 10). These data are expressed as mean ± SEM. *P < 0.05, **P < 0.01, ***P < 0.001, ****P < 0.0001 versus DMSO group.

When zebrafish exposed to varying concentrations of PFOS were treated with vitamin A, the grainy green patches on the fish noticeably reduced (Figure 5A). The identification of genes associated with apoptosis (Figures 5B–E) further suggests that vitamin A may prevent PFOS-induced alterations in zebrafish apoptosis-related genes.

### 3.7 Vitamin A alleviates anxiety-like behavior and social abnormalities in juvenile zebrafish caused by PFOS

**FIGURE 6:**
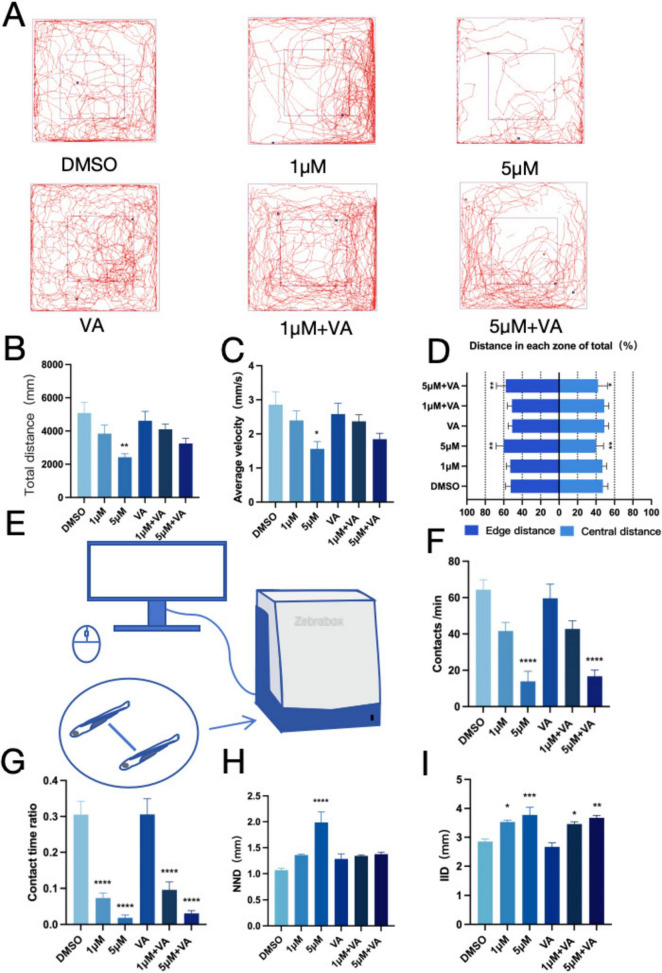
Zebrafish anxiety-like behavior and social interaction improved after vitamin A intervention. **(A)** Open field path map of zebrafish. **(B)** Total distance of zebrafish open field movement (*n* = 10). **(C)** Average speed of zebrafish open field (*n* = 10). **(D)** The proportion of distance between central and marginal areas of zebrafish (Edge area distance ratio = outer distance/total distance, central area distance ratio = inner distance/total distance) (*n* = 10). **(E)** Diagram of a zebrafish social interaction experiment. **(F)** Zebrafish contacts per min (Contact is defined as the distance between two fish ≤ one body length) (*n* = 12). **(G)** Zebrafish contact duration ratio (*n* = 12). **(H)** Zebrafish Minimum distance between two fish (*n* = 10). **(I)** Average distance between two zebrafish (*n* = 10). These data are expressed as mean ± SEM. *P < 0.05, **P < 0.01, ***P < 0.001, ****P < 0.0001 versus DMSO group.

We investigated the neurobehavioral effects of PFOS and vitamin A co-exposure in zebrafish to explore the potential role of vitamin A in reducing the neurotoxicity caused by PFOS exposure. We figured out that in the open field experiment (Figure 6A), zebrafish larvae showed significant improvements in both average speed (Figure 6C) and total movement distance (Figure 6B) following Vitamin A treatment. However, no significant changes were observed in the inner layer travel distance (Figure 6D). The social interaction assay (Figure 6E) revealed that vitamin A co-exposure partially restored the contact number (Figure 6F) and contact time (Figure 6G) compared to the PFOS-only group, though these parameters did not reach statistical significance. In the group behavior experiment, while the nearest neighbor distance (NND) showed a significant reduction (Figure 6H), the inter-individual distance (IID) exhibited a non-significant decreasing trend (Figure 6I), with values remaining comparable across experimental groups. According to the aforementioned research, Vitamin A may partially alleviate specific PFOS-induced behavioral and social challenges in zebrafish.

## 4 Discussion

Our attention has been drawn to PFOS, a perfluorinated compound characterized by its high content and considerable influence. A large number of studies have examined the bioaccumulation and refractory degradation of PFOS in the environment, and in recent years, its potential impact on neurodevelopment has become an important area of research. Findings from the Shanghai mother-and-baby cohort study indicate that fetal neurodevelopment was impacted following exposure to PFOS (Zhou et al., 2023). Additionally, Ayane Ninomiya et al. discovered that mice exposed to PFOS exhibit social dysfunction and decreased motor coordination—two important characteristics frequently linked to developmental disorders like autism spectrum disorder (ASD) (Ninomiya et al., 2022). These finding highlight the potential link between PFOS exposure and social behavior deficits, a critical component of neurodevelopmental conditions such as ASD. Non-placental animals like zebrafish provide an excellent platform for examining the neurotoxic consequences of PFOS in a more regulated and visible setting, particularly given the primarily exogenous nature of exposure. Moreover, the zebrafish model is especially useful for comprehending the wider effect of environmental pollutants on brain development and social function since it may be used to research social behavior abnormalities that are comparable to those seen in ASD.

Zebrafish are widely regarded as an ideal non-placental animal model, sharing approximately 70% genetic homology with humans (Howe et al., 2013). Direct observation of the negative effects of exposure to contaminants from the environment is possible due to the morphological and behavioral alterations of zebrafish models that are liable to such pollutants (Tao et al., 2022). This animal model is particularly useful for studying a range of brain functions, including novelty-induced responses, social behavior, and both spatial and social learning, which may be modeled by this animal model (Kirsten et al., 2018). The consequences of early-life exposure to PFAS have been increasingly studied using zebrafish models in recent years (Kim et al., 2021). Environmental concentrations of PFOS have been reported to range from 0.14 nM to 5 μM (Calafat et al., 2019; Cordner et al., 2019; Yu et al., 2020; Zeng et al., 2019). It has been previously documented that a PFOS concentration of 7.5 μM can be very harmful to zebrafish growth (Gaballah et al., 2020). Studies have shown that the EC50 of PFOS is 6.15 μM (Gui et al., 2023),Therefore, guided by toxicological design principles, we intended to investigate the neurodevelopmental alterations in zebrafish by selecting exposure doses of 1 μM (low) and 5 μM (high), in accordance with the toxicological design principles.

First, we evaluated the effects of PFOS exposure on zebrafish growth in general or the developmental toxicity of PFOS exposure. We assessed the zebrafish’s spontaneous movement at 24 hpf, and heartbeat at 48 hpf, measured their body length and head area at 72 hpf, and monitored the mortality and hatching of the infected fish. The results of our experiment clearly indicate that this dose did not significantly impair zebrafish growth in general. These findings align with previous reports on developmental toxicity, which identified a POD value of 11.42 μM (Christou et al., 2021; Min et al., 2023; Sant et al., 2021).

The impacts of developmental toxicity and motility remain key areas of research on PFOS toxicity in zebrafish. In this study, we investigate whether PFOS-induced neurodevelopmental toxicity impacts social interaction. To assess this, we examined the social and anxiety-related behaviors of the fish. Open field tests revealed that PFOS-exposed zebrafish displayed increased activity along the periphery compared to central areas. This suggests that the infected zebrafish were more likely to adhere to wall movement. Additionally, the decreased time spent in the central area relative to the marginal area indicates an increase in the tactile behavior of the zebrafish. Silvia Fuentes et al. observed that mice exposed to PFOS spent less time in the central area of the athletic field (Fuentes et al., 2007). In line with these findings, a study on anxiety-like behavior in *Oryzias latipes* demonstrated that anxious *Oryzias latipes* were more likely to remain in the periphery, avoiding the more brightly lit central area, thereby supporting our findings (Lucon-Xiccato et al., 2022).Although thigmotaxis in larval zebrafish is commonly used as a proxy for anxiety-like behavior (Schnörr et al., 2012), we acknowledge that developmental differences may influence behavioral interpretations. Our interpretation of thigmotaxis as indicative of anxiety-like behavior is based on observed locomotor patterns; however, future studies should integrate pharmacological challenges (anxiolytics) and neuroendocrine markers to confirm affective states. In the open-field test, both the total movement distance and movement speed of zebrafish were reduced, and the zebrafish exhibited a preference for edge zones, behaviors typically associated with anxiety-like responses. Zebrafish displaying Anxiety-like behavior were prone to increased freezing time (Mu et al., 2023), and freezing events were mostly concentrated in the central sensitive area of the open field. In the future, integrating the detection of cortisol content, histopathological analyses, and inflammatory factors to analyze the stress-related neural circuits and explore the specific links leading to social disorders becomes necessary. However, although some literature suggests that the aggregation behavior of zebrafish typically matures after 14 dpf (Dreosti et al., 2015; Martins et al., 2024), our findings indicate that PFOS exerts a preliminary impact on the approach pattern of zebrafish as early as 144 hpf. In social interaction and flock experiments, the social distance between zebrafish and other members of the same species increased, which was manifested as a decrease in total motor distance, a decrease in contact time and frequency, and an increase in adjacent distance between two fish, indicating social impairments. Similarly, Mu et al. evaluated the behavioral characteristics of zebrafish infected with bisphenol A and observed that zebrafish treated with bisphenol A exhibited significant social behavioral deficits and increased social distance from conspecifics (Nunes et al., 2021). A study by Bai et al. also supports the hypothesis that environmental endocrine disruptors can cause symptoms of ASD-like social disorder in zebrafish (Bai et al., 2023). Although the research results indicated potential social barriers, the observed differences might also be influenced by non-social factors, such as sensorimotor or environmental cues. It can be detected through motion tracking analysis. Larvae are based on adjacent interaction patterns, and these early patterns may serve as precursors of social behavior, although further research is needed to confirm this.

We examined the genes involved in the development of the nervous system to better understand the altered behavioral activities. Our findings revealed that PFOS had an impact on early neurogenesis (*elval3*) and the central nervous system (*bdnf, gfap, and mbpa*). DA system alteration in zebrafish may occur due to PFOS exposure (Wang J. et al., 2022; Wu et al., 2022; Zhang Y. et al., 2023). To investigate this alteration, we searched for DA-related genes and DA content. We discovered that DA production (*th1, th2*), transport (*dat*), and related receptors (*drd2a, drd3, drd4a, and drd1*) were all altered, albeit to different degrees. TH serves as the rate-limiting enzyme in DA biosynthesis. The elevated mRNA levels of its isoforms (th1 and th2) may indicate augmented synthetic capacity. Some studies have also shown that PFOS may disrupt DA homeostasis through multiple pathways, inhibiting VMAT2-mediated vesicle storage, leading to abnormal accumulation of cytoplasmic DA, and accelerating oxidative metabolism (Patel et al., 2016). In a study by Foguth et al., they found that PFOS exposure increases the DA turnover rate, reflecting enhanced compensatory metabolic clearance. Therefore, the coupling imbalance of synthesis-storage-metabolism may represent the core mechanism underlying the abnormal DA pathway caused by PFOS exposure. To precisely delineate PFOS’s impact on dopaminergic pathways, future studies should incorporate pharmacological interventions (α-methyl-p-tyrosine for TH inhibition) combined with advanced neuroimaging techniques like positron emission tomography (PET). Such approaches will enable quantitative assessment of DA turnover kinetics and spatiotemporal neurotransmitter dynamics. This study reveals the time-dependent characteristics of PFOS neurotoxicity. The difference between the subthreshold changes of Drd1/Drd3 expressions observed in zebrafish exposed to high concentrations of PFOS at 144 hpf (Figure 2) versus 168 hpf (Figure 4) indicates that the effect of PFOS on DA receptors may be related to the prolonged exposure time. This dynamic response may be related to the bioaccumulation of PFOS and its progressive interference with transcription factors. Previous studies have indicated that DA receptor expression is more sensitive to environmental stress in the later stages of neurodevelopment (Barreto-Valer et al., 2012; Paiva et al., 2020). The significant upregulation of PFOS observed in the exposed group (168 hpf) in this study further validates this rule, suggesting that the time window effect of the developmental stage should be comprehensively considered when evaluating the neurotoxicity of PFOS. Simultaneously, a rise in the level of DA was detected; this alteration in the dopaminergic system would lead to apoptosis (Quintero-Espinosa et al., 2023), an essential stage in normal neurodevelopment. This study revealed that exposure to PFOS enhanced the formation of apoptotic bodies. The experimental data revealed that PFOS exposure significantly up-regulated the expression of pro-apoptotic gene bax and apoptotic executive protein gene caspase 3/8/9 (*p* < 0.05). Additionally, acridine orange (AO) staining revealed increased apoptosis. This phenomenon suggests that PFOS exposure can cause changes in apoptosis-related genes. However, whether these toxic effects are specifically mediated through the activation of apoptotic signaling networks rather than other programmed cell death (such as necrosis or autophagy) or non-death-related pathways (such as proliferation or differentiation) needs to be further verified. These findings are consistent with those of earlier research (Chen et al., 2012; Dong et al., 2015; Gao et al., 2024; Ge et al., 2016; Shi et al., 2024; Tang et al., 2022).

We hypothesize that treating zebrafish with a readily supplemented nutrient that may control DA may lessen the neurotoxicity of PFOS exposure by reducing the modification of DA, as this may be a significant mechanism for the neurotoxicity of PFOS. Retinoic acid—a fat-soluble micronutrient (VanBuren and Everts, 2022), and metabolite of vitamin A, is essential for brain development (Hernández-Pedro et al., 2014; Zhong et al., 2013). Inadequate consumption of vitamin A during pregnancy may result in brain abnormalities, heightened vulnerability to deviant behavior, neuropsychiatric diseases, and cognitive alterations. Studies have shown that weekly administration of vitamin A for 6 months can significantly improve social functioning in children aged 3–8 years with vitamin A deficiency (Lai et al., 2021). Our findings suggest that vitamin A was effective in reducing both apoptosis and abnormal DA levels in PFOS-exposed zebrafish, which may translate into changes in their proximity-based interactions. Ultimately, vitamin A improved the neurotoxicity and early homospecific behavioral changes induced by perfluorooctane sulfonate (PFOS) in zebrafish larvae by regulating dopaminergic signaling and apoptosis.

Studies have shown that PFOS exposure may disrupt the normal nervous system development in zebrafish through multiple pathways. Notably, the expressions of apoptosis-related proteins Caspase and Bax were significantly up-regulated in the PFOS treatment group, suggesting that the apoptosis or activation of the programmed death process of nerve cells, which may directly lead to the reduction of the number of nerve cells or functional damage. Concurrently, the abnormal expression of key genes of the DA pathway (such as DA receptors and transporters) suggests that PFOS may affect the formation and regulation of neural circuits by interfering with dopaminergic signaling. Further behavioral analysis revealed that juvenile zebrafish in the exposed group exhibited decreased motor activity and contact among conspecifics, both of which are closely associated with neuromotor coordination and cognitive function. Based on the above results, it can be inferred that PFOS may induce nerve cell apoptosis, disrupt DA system homeostasis and cause behavioral dysfunction, and eventually lead to neurodevelopmental abnormalities in zebrafish. These findings provide an important experimental basis for the study of the neurotoxic mechanism of PFOS. Moreover, the intervention of vitamin A mitigates the changes in apoptosis-related genes and DA signaling pathways, suggesting that vitamin A can be used as a protective agent against PFOS-induced neurodevelopment.

In this study, zebrafish were employed as the animal model. Although zebrafish offer numerous advantages in developmental biology and disease model research, inherent physiological and metabolic differences exist compared to mammals, including humans. Consequently, the applicability of the study’s findings to humans may be somewhat limited. Future research could benefit from utilizing mammalian models to better simulate the protective effects of vitamin A on the nervous system, thereby validating its efficacy under conditions more closely aligned with human physiology. Additionally, the optimal protective dosage of vitamin A warrants further investigation, particularly regarding its long-term impact on the nervous system and its protective effects in adulthood.

## 5 Conclusion

In conclusion, this study demonstrates that PFOS exposure induces neurotoxicity in zebrafish, characterized by DA system dysregulation, apoptosis, and behavioral alterations including reduced proximity to conspecifics and increased anxiety-like behavior. Vitamin A supplementation significantly attenuated PFOS-induced neurochemical changes (e.g., DA elevation and apoptosis-related gene upregulation) and partially restored proximity maintenance deficits, particularly in the low-dose PFOS group (1 μM PFOS + VA). However, in the high-dose PFOS group (5 μM PFOS + VA), aggregation parameters (contact frequency and IID) remained impaired despite VA treatment, suggesting a dose-dependent limitation of VA’s protective efficacy. These findings support the utility of the zebrafish model for studying PFOS-induced neurotoxicity and highlight the DA system as a critical target for intervention. Further research is needed to optimize VA dosing strategies and explore combinatorial therapies to fully reverse high-dose PFOS-induced aggregation deficits. Although, these results position vitamin A as a protective agent against PFOS-induced neurodevelopmental perturbations, its efficacy in adult social/group behavior recovery requires further investigation.

## Data Availability

The original contributions presented in the study are included in the article/Supplementary material, further inquiries can be directed to the corresponding authors.
