# Supplementary figures and images for: Vitamin A attenuates PFOS-induced neurotoxicity and alters early proximity patterns to conspecifics in zebrafish larvae
Source: Front Behav Neurosci. 2025 Jun 5;19:1564694. doi: 10.3389/fnbeh.2025.1564694 (PMC12176739; doi:10.3389/fnbeh.2025.1564694)

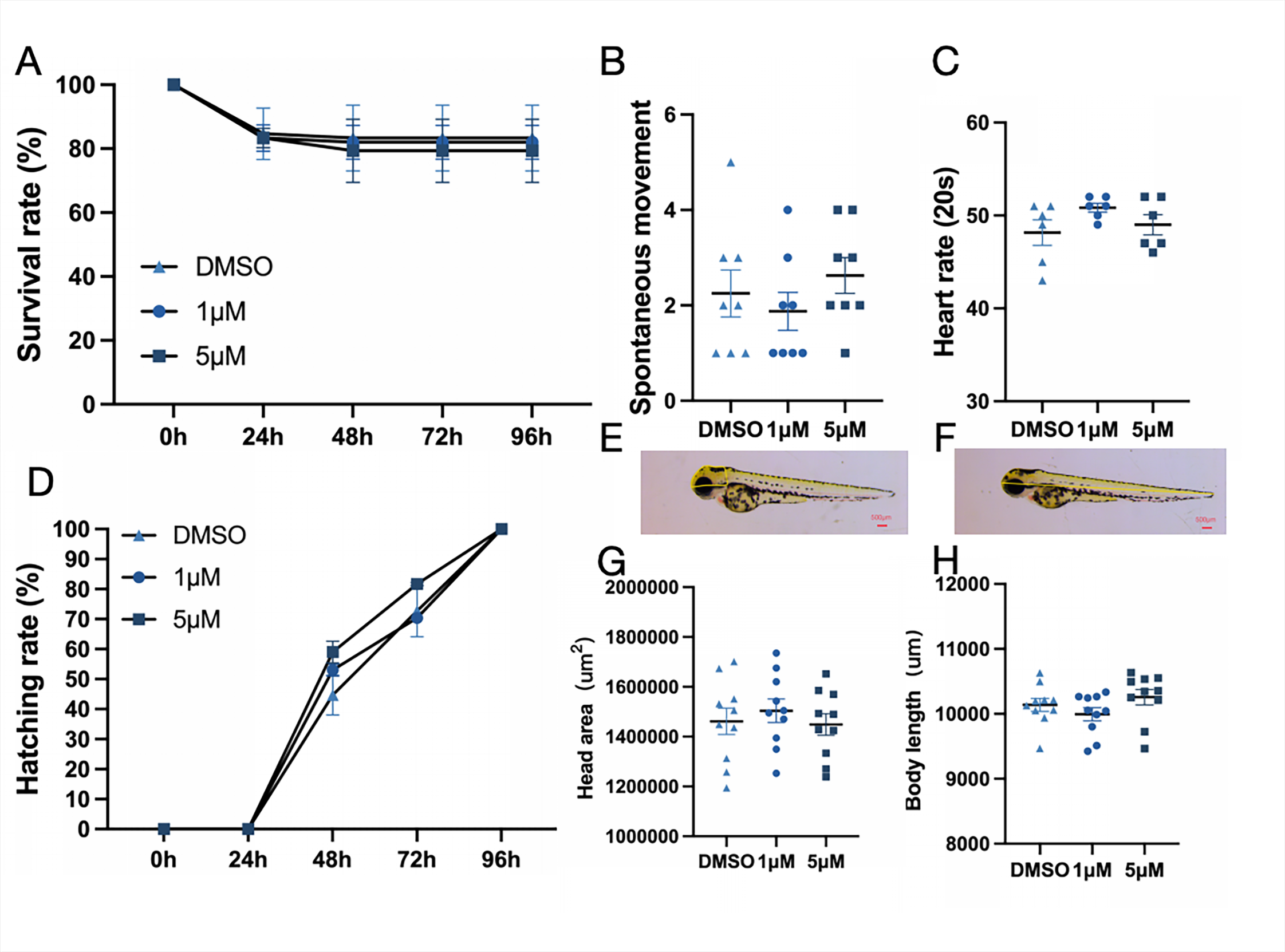

Supplement: Supplementary file 1 [file Image_1.tif]
